# Analytical Methods for the Determination of Neuroactive Steroids

**DOI:** 10.3390/biom11040553

**Published:** 2021-04-09

**Authors:** Michal Kaleta, Jana Oklestkova, Ondřej Novák, Miroslav Strnad

**Affiliations:** Laboratory of Growth Regulators, Faculty of Science and Institute of Experimental Botany of the Czech Academy of Sciences, Palacký University, Šlechtitelů 27, CZ-78371 Olomouc, Czech Republic; michal.kaleta@upol.cz (M.K.); novako@ueb.cas.cz (O.N.); miroslav.strnad@upol.cz (M.S.)

**Keywords:** immunoassay, mass spectrometry, metabolomics, neuroactive steroids, steroid

## Abstract

Neuroactive steroids are a family of all steroid-based compounds, of both natural and synthetic origin, which can affect the nervous system functions. Their biosynthesis occurs directly in the nervous system (so-called neurosteroids) or in peripheral endocrine tissues (hormonal steroids). Steroid hormone levels may fluctuate due to physiological changes during life and various pathological conditions affecting individuals. A deeper understanding of neuroactive steroids’ production, in addition to reliable monitoring of their levels in various biological matrices, may be useful in the prevention, diagnosis, monitoring, and treatment of some neurodegenerative and psychiatric diseases. The aim of this review is to highlight the most relevant methods currently available for analysis of neuroactive steroids, with an emphasis on immunoanalytical methods and gas, or liquid chromatography combined with mass spectrometry.

## 1. Introduction

Neuroactive steroids (NASs) can be characterized as substances of steroid origin that can have effects on the nervous system [1]. They include hormonal steroids which originated in the peripheral glands, steroids locally synthesized by neurons and glial cells, and synthetic steroids that modify the activity of the nervous system [2].

**Figure 1:**
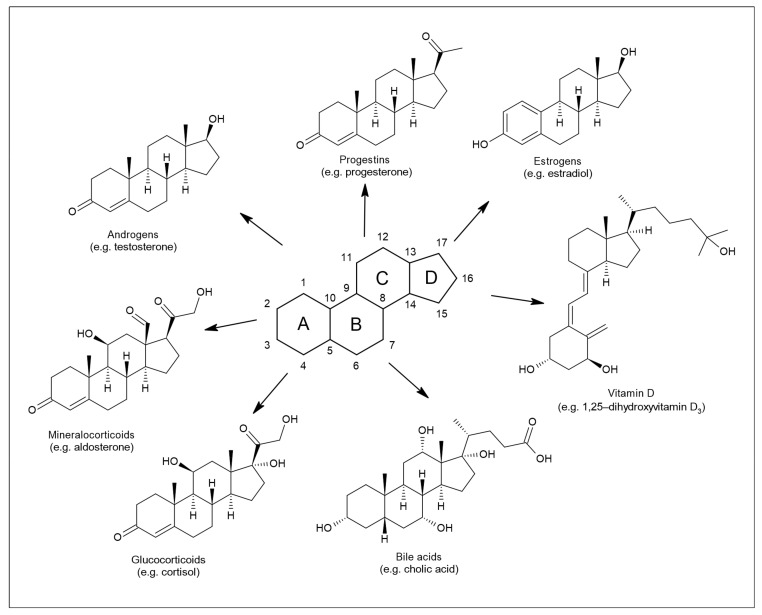
Structure of sterane and seven principal classes of steroid substances.

Their core structure, as for other steroids, is represented by sterane or cyclopentanoperhydrofenanthrene [3] (Figure 1). Members of this group, such as progesterone, estrogens, testosterone, dehydroepiandrosterone (DHEA), or cortisol, are involved in shaping the structure and function of the central nervous system throughout the life cycle [4]. The nervous system is affected by both endogenously synthesized NASs and steroids of exogenous origin [5]. The first location for NAS steroidogenesis is the peripheral endocrine gland. However, the biosynthesis of these substances can also occur directly in the central and peripheral nervous systems, based on which this specific subgroup is referred to as neurosteroids [6]. Steroids of exogenous origin include substances prepared synthetically. The regulation of many processes in the body is based on the ability of NASs to interact with different types of receptors [7]. In particular, these are γ-aminobutyric acid (GABA) receptors, N-methyl-D-aspartate (NMDA) receptors, voltage-gated calcium channels, voltage-dependent anion channels, serotonin receptors, microtubule-associated protein 2, and others.

**Figure 2:**
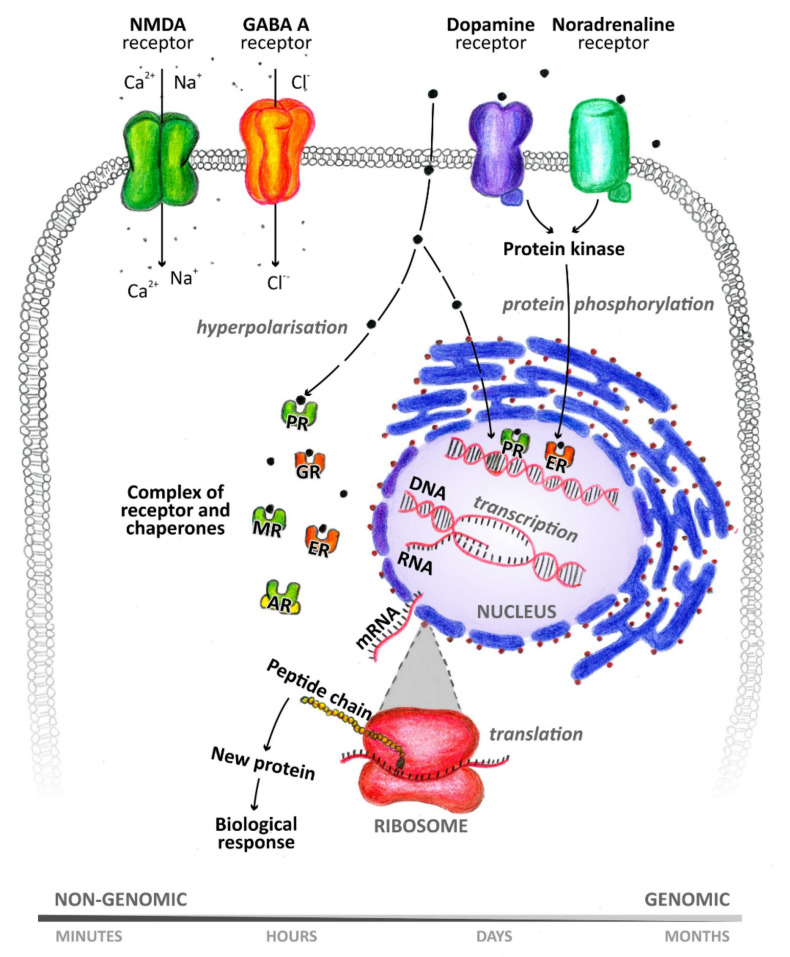
Genomic and nongenomic mechanism of neuroactive steroid action. PR: progesterone receptor, GR: glucocorticoid receptor, MR: mineralocorticoid receptor, ER: estrogen receptor, AR: androgen receptor.

Many metabolites of sex hormones and some stress hormones act in the central nervous system through the so-called nongenomic mechanism, the effect of which is manifested within a period ranging from a few milliseconds to seconds [8] (Figure 2). These rapid nongenomic effects are made possible due to the interaction of NASs with ion channels and membrane receptors [9]. In contrast, when steroids interact with nuclear or cytoplasmic receptors, the resulting effect occurs after a longer period of time [10]. This is because these intracellular receptors function as transcription factors and are involved in gene expression [11]. NASs can thus affect the function of neurons, as well as other cells of the brain, such as astrocytes, microglia, oligodendrocytes, and endothelial cells [1]. NASs are likely involved in the regulation of neurogenesis, neuron survival, neuritogenesis, glial cell differentiation, myelin formation, and synaptic plasticity. Their neuroprotective effects and their ability to suppress nerve tissue inflammation are also described. For a wide range of neurological and psychiatric diseases, such as schizophrenia, epilepsy, depression, or multiple sclerosis, these substances play an important role in their pathology and therapy [11]. Hormonal differences between men and women are noticeable under both physiological and pathological conditions [1]. It has already been shown that neurodegenerative diseases, such as multiple sclerosis, can affect the levels of circulating NASs [12]. Monitoring these changes in blood plasma or cerebrospinal fluid (CSF) can serve as a warning signal (biomarkers) to draw attention to pathological processes taking place in the nervous system.

Knowledge of NAS formation and their correct detection can be used to prevent and treat some neurodegenerative and psychiatric diseases. The aim of this article is to provide an overview of the most important methods for NAS analysis, with an emphasis on immunoanalytical methods and gas (GC), or liquid chromatography (LC) combined with mass spectrometry (MS).

## 2. Determination of Neuroactive Steroids

Efforts to quantify steroid hormones using colorimetry-based methods have existed since the 1930s [13,14,15]. One of the first methods used to define and quantify hormonal activity was also a bioassay [16]. The so-called whole animal in vivo bioassays used before the introduction of radioimmunoassays (RIAs) were arduous and insensitive [17]. Despite large advances in molecular–genetic methods (e.g., PCR, DNA sequencing), determining the metabolic profile of NASs is still important [18]. The information obtained can be used to diagnose, but also to monitor, the course of a disease. Immunoassay- (IA) and MS-based methods are currently used to monitor steroid hormone levels [19]. They provide higher sensitivity, specificity, and reliability. RIAs and direct immunoassays (DIA) dominate among IA approaches.

### 2.1. Types of Biological Matrices

To determine steroid hormones in clinical practice, blood serum and plasma samples are mainly used [20,21,22,23,24,25]. Moreover, saliva can be an alternative biological material. The advantages of this type of sample are that it is noninvasive, less stressful, and easier to collect compared to blood. Saliva sampling can also be performed in a home environment. In addition, there is a correlation between levels of unconjugated steroid hormones in saliva and levels of their unbound fraction in blood serum. The final levels may differ, which is a consequence of steroid metabolism in the salivary glands. The ratio of cortisol concentrations in saliva and blood serum is around 1:20 and, for testosterone and estradiol, it approximates to 1:90. Furthermore, steroids can be analyzed in other biological matrices, such as urine, CSF, hair, or nails [26,27,28,29,30,31,32]. Urinary steroid profiling has been an integral part of the diagnosis of steroid metabolism disorders for several decades (since the 1960s) [33]. Great interest is focused on the analysis of steroids in human CSF [12,32,34,35,36]. CSF is the only matrix that can be obtained from living donors and it allows for the monitoring of brain metabolism. As mentioned above, the nervous system is a source of neurosteroids. Central neurosteroids are studied as potential biomarkers of various cognitive disorders (e.g., dementia, depression). Extensive research in and use of this matrix are complicated by its limited availability, due to the complexity of collection (usually lumbar puncture), the limitations of its indication in healthy individuals (very small participant groups), as well as its limited sample amount for analysis. Due to the trace levels of neurosteroids in CSF, great demands are also placed on analytical techniques. The interested reader is referred to the cited reference for detailed description of CSF analysis [34].

### 2.2. Factors Affecting Steroid Hormone Levels

The results of NAS analysis are affected by several important factors, such as age, sex, and, of course, sampling time [19]. A natural decrease in the levels of certain steroids in relation to male age has been found. A similar trend can be observed for women; however, hormonal changes also occur during the menstrual cycle. A decrease in testosterone levels with increasing age in men and women has also been determined in saliva [24]. Levels of some steroid hormones in humans fluctuate during the day (e.g., cortisone, cortisol, corticosterone, 11-deoxycortisol, androstenedione, 17α-hydroxyprogesterone, DHEA, testosterone) [37,38]. Therefore, it is important to take samples under standardized conditions. Boyce and co-workers (2004) published different reference ranges for serum testosterone levels in men depending on the time of day [39]. The range for morning testosterone concentrations is 10.07–38.76 nmol.L^−1^ for men under 40 years, and 7.41–24.13 nmol.L^−1^ for men over 40 years of age. The evening ranges are then 6.69–31.51 nmol.L^−1^ and 6.46–21.93 nmol.L^−1^, respectively. Salivary testosterone levels for men and women even differ depending on the season [24]. Keevil and co-workers (2017) found the lowest levels of salivary testosterone for men, and the highest for women, in the summer (June–August). In addition to age, sex, and time of day, total testosterone levels are also affected by various pathological conditions [40].

## 3. Analysis of Neuroactive Steroids by Immunoassays

### 3.1. History of Immunoassays

The year 1959 was ground-breaking because Rosalyn S. Yalow and Solomon Berson published their immunoanalytical method for determining insulin in human blood plasma using radioisotope antigen labelling [41]. The discovery of RIA revolutionized the possibilities of determining the levels of hormones, drugs, vitamins, viruses, tumor antigens, etc. [42,43]. In 1969, Abraham described an RIA-based method allowing 17β-estradiol to be determined [44]. After this first RIA, applications allowing the determination of estradiol and IAs were developed to determine other steroid hormones, such as testosterone or progesterone [45].

The advantages of RIAs include their accuracy and reliability [19]. Radioactive markers, such as ^125^I, ^32^P, or ^14^C, are used as indicators in this case [46]. Because radioactivity is used, it is necessary to ensure the safety of laboratory personnel, to establish specialized facilities, and to ensure the disposal of radioactive waste [47]. Although this method of analysis of both steroids and sterols is highly sensitive, it is not specific enough [48]. In addition, it is necessary to define the established analytes before self-analysis.

### 3.2. Preparation of Antibodies

Similar to other low molecular weight substances, steroids are not immunogenic, which is contrary to chemical compounds with a molecular weight over 1000 kDa that are usually immunogenic [46,49]. As with all other low molecular weight haptens, the immunogenicity of steroids can be increased by conjugation to a high molecular weight carrier (usually a protein carrier such as serum albumin, ovalbumin, or keyhole limpet hemocyanin). It is generally accepted that, in the preparation of highly specific antisera, the carrier protein should be conjugated to the steroid molecule via sites on its cyclohexane rings B or C [18]. The location of the chemical bond between the high molecular weight carrier and the steroid molecule most significantly affects the specificity of the antiserum obtained.

### 3.3. Enzyme and Direct Immunoassay

Over time, methods using indicators other than radioisotopes, in particular enzymatic, fluorescent, or chemiluminescent labeling, have also emerged [45]. At the turn of the 1960s and 1970s, the idea arose to use enzymes (e.g., alkaline phosphatase, glucose oxidase, and horseradish peroxidase) to label antigens or antibodies, which proved to be feasible and served as a basis for the development of the enzyme immunoassay (EIA) [47]. However, compared to RIA, sensitivity may be reduced [43].

In the 1980s and 1990s, IAs spread to clinical and laboratory practices, which was consistent with efforts to simplify them, increase sample throughput, and lower the price [50]. Due to the health risk associated with the use of radioisotopes, as well as time requirements and complexity, RIAs in clinical laboratories have been gradually displaced by DIA methods using enzyme labeling [19]. The advantage of this method lies in its security, lower financial costs, commercial convenience, and the possibility of automation. Although testing on the analyzer platform is simple, fast, high capacity, commercially friendly, automated, and affordable, the introduction of DIAs has been associated with a deterioration in testing performance [51]. To increase specificity, more efforts are needed to design individual kits and antibodies. As a result of omitting the extraction step and chromatography in automated IAs, specificity decreased [45].

### 3.4. Antibody Specificity

Routine diagnostic laboratories mainly use immunoanalytical techniques [33]. However, there are limitations to their use. Antibody specificity is a property that expresses its ability to distinguish between the antigen against which it was produced and any other antigens present [52]. The specificity of an antibody can be defined by cross-reactivity, which indicates the ability of the antibody to cross-react with antigens other than the immunogen. Thus, the results provided by IAs may be overestimated if the level of cross-reactivity is high [53]. Cross-reactivity is usually a result of the presence of structurally similar analytes, such as other endogenous substances, pharmaceuticals, and natural products, in addition to metabolites of these compounds, which may cross-react with antibodies [54]. For example, many direct platforms created for testosterone analysis interfere with dehydroepiandrosterone sulfate [55,56]. Interference with the synthetic steroid, mifepristone, used as a contraceptive, has been observed in some commercially available EIAs for the determination of estradiol and testosterone [57]. False overestimation of the result may occur even in the presence of substances with low cross-reactivity, but at a concentration higher than the concentration of the analyte. For this reason, an extraction step and/or chromatographic separation can be included before IAs, ultimately making these techniques more accurate and sensitive compared to DIAs [40]. Extraction and chromatographic separation allow removing interfering substances and separation of cross-reacting steroids from the analyte.

### 3.5. High-Dose Hook Effect

Another problem associated with IA may be the so-called high-dose hook effect, which leads to a false undervaluation of the analysis results [58,59,60,61]. This effect has been observed in the “sandwich” IAs. There is an interference between antigens in high concentration and IA. In some cases, the concentration of the analyte reaches a certain point, the system saturates, and the formation of “sandwiches” is prevented, thus the signal reduces. This effect has already been observed in the steroid hormones aldosterone, testosterone, and 17-hydroxyprogesterone. Insufficient recognition of this phenomenon may have a negative effect on the patient because it can lead to wrong diagnosis and improper therapy. This problem can be solved by a change of ratio between antigen and antibody, either by modifying IA or diluting the analyzed sample.

### 3.6. Positives of Immunoassays

IAs are still a widely used method for the quantification of steroid hormones, mainly due to the high throughput of samples, simple and fast execution, and relatively low cost [53,62]. IAs such as RIA and the enzyme-linked immunosorbent assay (ELISA) are commonly used in laboratories for the determination of estrogen metabolites in blood or urine because of their efficiency and low cost [63]. Commercially available kits, which can be used manually and also enable automation, are often used in different laboratories [64]. Unlike MS, working with IAs does not require highly qualified personnel, and their setup and execution is relatively easy [18]. It is important to ensure that IAs are only used for the purposes for which they were originally developed. It is also necessary to take their limits into account.

### 3.7. Limitations of Immunoassays

The biggest limitation of these methods is that they focus on only one analyte, meaning that each analyte requires its own IA [17]. If it is necessary to analyze more steroids in a sample, it is necessary to use the appropriate IA for each analyte, which is associated with higher financial and time costs and, of course, higher consumption of the sample [17,65]. Therefore, each sample must be aliquoted for individual testing.

Despite the widespread use of IAs, the results that these methods provide may sometimes be misleading [58]. A general problem with IAs is their lack of sensitivity and specificity of antibodies, in addition to their sensitivity to matrix effects and interferences [19]. The specificity of IAs is also questionable, especially when determining low levels of steroid hormones [66]. There is no sufficient congruence between the different platforms because different IAs use antibodies directed against different epitopes to quantify the same analyte [58]. Another problem may be the presence of endogenous autoantibodies or antibodies. Unfortunately, commercially available tests are not properly validated by the manufacturer, especially concerning their sensitivity, accuracy, precision, and specificity, and can thus provide unreliable results [64]. In their paper, Stanczyk and co-workers (2003) examined the reliability of commercial diagnostic IAs that are used to quantify serum testosterone and estradiol levels. This study evaluated nine commercial IA kits used for estradiol determination working on the principles of RIA, EIA, and chemiluminescent IA (CIA). The method used in most cases was DIA without the purification step. To determine the reliability of testosterone determination kits, four different direct RIAs and CIAs were tested. The results were compared with those obtained using conventional RIA, which included an extraction step and chromatographic separation. In general, the authors noted large differences in the determined levels of these hormones in the samples when they used kits from different manufacturers to quantify them.

Faupel-Badger and co-workers (2010) compared the determination of selected steroid hormones using indirect RIA and ELISA with LC–MS/MS (liquid chromatography–tandem mass spectrometry), and also observed these discrepancies [63]. In this work, urine samples from premenopausal and postmenopausal women were used to analyze steroid hormones. The results showed that the absolute concentrations of estrone, estradiol, estriol, 2-hydroxyestrone, and 16α-hydroxyestrone, which were provided using immunoanalytical methods, were 1.6–2.9 higher in premenopausal women and 1.4–11.8 times higher in postmenopausal women than the concentrations determined by LC–MS/MS. This overestimation may be due to the cross-reactivity with other estrogen metabolites. LC–MS/MS measurements highly correlated (Spearman r (rs) = 0.8–0.9) with RIA and ELISA measurements in premenopausal women. However, only a slight correlation was observed in postmenopausal women (rs = 0.4–0.8).

The use of these tests to determine low levels of steroid hormones that are typical for postmenopausal women and children can also be problematic [33]. According to Taieb and co-workers (2003), the use of IAs to determine the low and very low testosterone concentrations (0.17–1.7 nmol.L^−1^) expected in women and children is not sufficiently reliable [67]. In their study, they compared the determination of testosterone in the blood serum of 50 men, 55 women, and 11 children using different DIAs (eight non-isotopic methods and two RIAs) and isotope-dilution GC–MS. A total of 7 out of 10 IAs provided an average of 46% higher testosterone levels in women’s samples compared to GC–MS analysis. In men, by contrast, IAs results were, on average, 12% lower.

Huhtaniemi and co-workers (2012) compared the determination of testosterone and estradiol in men’s blood serum using a commercially available electrochemiluminescent IA platform (ECLIA) (Roche Diagnostics E170) with GC–MS determination [62]. Their results indicated that the testosterone concentrations measured by IA showed a high correlation with the concentration determined by MS over a wide concentration range. In the case of the hypogonadal range (<11 nmol.L^−1^), the correlation was less significant; however, this IA platform is sufficient to detect the subnormal testosterone concentrations observed in men with hypogonadism. Overall, weaker correlations between IA and MS were observed in estradiol assays. IAs in this case are only suitable for the detection of high estradiol concentrations in men (>120 pmol.L^−1^).

## 4. Analysis of Neuroactive Steroids by Mass Spectrometry

In recent years, MS has also become a method used for steroid analysis due to its high sensitivity and specificity [45]. However, IAs (those with satisfactory specificity and sensitivity) should not be completely replaced, because both approaches may complement each other. Immunoanalytical methods are still preferred in steroid analysis, mainly due to their simplicity, lower costs, and availability of commercial kits and reagents that do not require special staff skills [51]. The implementation of MS is very expensive, technically demanding, and requires special instrumentation. However, manufacturers have made significant efforts to develop more user-friendly MS technologies that can also be used in clinical laboratories, and which are able to perform routine analyses with high robustness and throughput.

Steroid analysis often uses chromatographic methods which, however, yield only limited information about separated compounds such as retention times [18]. For correct characterization, these techniques should be coupled with a suitable detection system. The ideal tool that allows analysis of a wide spectrum of compounds is the MS detector. The unequivocal advantage of quantification of steroid hormones by MS is the ability to analyze several analytes simultaneously in a single injection with high selectivity, sensitivity, accuracy, and precision [66].

The combination with chromatography is particularly advantageous due to its versatility and high separation strength [68]. Another advantage is the availability of various chromatographic techniques and the number of separation mechanisms that can be used. Better separation power can be achieved using comprehensive two-dimensional chromatographic technologies (GC × GC, LC × LC).

### 4.1. Internal Standards

Quantitative MS analyses are mainly based on the use of internal standards, which are compounds of either similar structure, analyte analogues, or stable isotope-labeled (SIL) standards bearing, for example, ^13^C, ^15^N, ^17^O, or ^2^H [69,70]. The use of SIL standards has become increasingly dominant in recent years. In general, the use of internal standards makes it possible to compensate for analyte losses during sample processing and purification [71]. They also eliminate the variability of injected volumes and the MS signal response due to the effect of sample matrices on ionization. Moreover, loss of some analytes may occur, e.g., due to incomplete extraction, derivatization protocol, or degradation of analytes during storage [72]. During daily operation, fluctuations in the temperature and pressure of the ion source can also affect the MS ionization efficiency of the MS analyzer [69].

However, even the use of SIL standards is not always ideal [73]. SIL internal standards are more costly to prepare and may not be commercially available in all cases. Some deuterated internal standards also showed discrepancies in retention times and recoveries of analytes versus SIL [74]. The purity of the SIL standards used is also important [69]. If they are not available or are too expensive, the use of structural analogues may be an alternative solution [69,70].

### 4.2. Gas Chromatography–Mass Spectrometry

#### 4.2.1. Introduction and History of GC–MS

History of the use of GC for steroid separation dates back to the early 1960s [75]. In 1964, Eneroth and his team published a paper focused on identification and quantification of neutral steroids in human feces using an analytical tool combining GC and MS [76]. Because both the ion source and the MS analyzer required a high vacuum, the MS was originally combined with GC separation due to the gas phase operation and the relative ease of coupling [65].

#### 4.2.2. Gas Chromatography

GC is a separation technique which separates components of a mixture based on their different affinity to stationary phase of chromatography column [77,78]. Due to the relatively high molecular weight of steroids, a high temperature is required (usually above 200 °C) during gas chromatographic separation [79]. After evaporation, the sample is batched into the mobile phase, in this case, carrier gas, most commonly helium or hydrogen [77,78]. Separated components exiting the chromatography column are then detected using a detector (e.g., mass spectrometer). The chromatography columns made of fused silica allow an easy coupling with MS. These columns with conventional stationary phases, based on polysiloxane or polyethylene glycol, are still commonly used [80].

#### 4.2.3. GC–MS Sample Preparation: Hydrolysis and Derivatization

In the case of analysis of polar, thermolabile, and/or nonvolatile analytes, it is necessary to use a derivatization step [81]. Steroids are nonvolatile compounds and can decompose during analysis due to high temperatures [65]. Derivatives obtained by chemical modifications are more hydrophobic, volatile, and temperature-stable compared to original steroids. Prokai-Tatrai and co-workers (2010) developed a gas chromatography–tandem mass spectrometry (GC–MS/MS) method for the analysis of 17β- and 17α-estradiol and estron in human blood serum [25]. Sample preparation included liquid–liquid extraction (LLE) and one-step derivatization with *N*-(trimethylsilyl)imidazole. Nilsson and co-workers (2015) chose a derivatization technique based on steroid oximation and esterification to profile seven sex steroid metabolites in rodent blood serum [66]. These modifications were made to achieve an exceptionally high sensitivity using a triple quadrupole operating in selective multi-reaction monitoring (MRM) mode.

During derivatization, chemical changes in analytes occur, associated with changes in their physical–chemical properties [82]. The derivatization step improves the volatility and thermal stability of the analytes [48,83]. This step leads to a better chromatographic separation and an increase in the sensitivity of the method [82]. Derivatization does not only apply to GC–MS, because it can also be used in the preparation of samples for LC–MS/MS [32,53]. Despite these positives, there may be discrimination of analytes in the sample during derivatization (different derivatization efficiencies, loss of compounds), contamination, and the formation of by-products [80]. Moreover, derivatization can also lead to isomer mixtures of derivatives [65].

The low throughput of GC–MS is caused by time-consuming sample preparation [80]. The need to examine a large number of samples, e.g., in population studies, is a problem because this method is characterized by higher time demand due to the need for derivatization, and also requires higher sample volumes [53].

If steroid conjugates (e.g., sulphates, glucuronides) are present, enzymatic or chemical hydrolysis of charged groups can also be performed [65]. The GC–MS tools require cleavage of the sulphate group, in addition to subsequent derivatization [84,85]. In the case of GC–MS analysis of sulphate conjugates, increases in signals may occur due to the presence of other conjugates, especially glucuronides. This is caused by the fact that commercially available sulfatases often also show glucuronidase activity; furthermore, chemical hydrolysis of the sulphate group is also not specific. However, the LC–MS technique allows for the analysis of intact sulphate steroid conjugates without any modification.

#### 4.2.4. GC–MS Ionization Techniques

The GC system coupled with MS is one of the most universal, standard, and used analytical tools [86,87]. This approach has been used for the analysis of steroid metabolites for several decades. Due to the high reproducibility, sensitivity, and availability of mass spectrum databases, GC–MS and GC–MS/MS techniques have often been used in these studies [72]. The most common ionization technique applied in metabolic studies based on GC is electron impact ionization (EI) [81,88,89]. This hard ionization technique can help to achieve the reproducible fragmentation of molecules [18,81]. Using EI and GC–MS, several spectral libraries and databases are available. Chemical ionization (CI) is another ionization technique optimal for steroid analysis, and it is commonly used due to less fragmentation of analyte molecules and higher occurrence of parent ions. Polet and co-workers (2016) used CI in their study focused on the analysis of anabolic steroids [90]. The abovementioned EI is often used in steroid analysis [88,91,92,93,94]. For example, Hill and co-workers (2019) described a method for the determination of 100 endogenous steroids in human blood serum by EI combined with GC–MS/MS [95].

#### 4.2.5. GC–MS and Their Applications

GC–MS is a tool that can be used in both targeted and untargeted analysis approaches [80]. Compared to LC–MS, this analytical technique provides higher sensitivity, resolution, reproducibility, reliability, and relatively low cost [72]. For example, profiling of steroids in urine by GC–MS and GC–MS/MS is a suitable tool for the discovery of new steroids, their characterization, and the acquisition of new knowledge useful for the diagnosis of metabolic disorders [51,93]. Due to its excellent chromatographic resolution, GC–MS allows for the identification of new therapeutic metabolites [45].

GC–MS systems work in both full scan mode and selected ion mode (SIM) [33]. GC–MS in scanning mode is a suitable tool for the untargeted profiling of steroid hormones, the discovery of new compounds, and the study of metabolic pathways. Spectral databases are appropriate tools for identifying unknown compounds [18]. Using SIM acquisition, GC–MS analysis can achieve higher sensitivity because of noise reduction [65]. Due to the selectivity and higher sensitivity of this mode, it is preferably used for quantification [18]. However, high background noise is a problem when analyzing very low concentrations of analytes using the classical single-stage GC–MS [96,97].

Further development has recently led to the connection of GC with tandem mass spectrometers [95]. GC–MS/MS can be used to eliminate background interference and increase overall sensitivity and specificity [96,97]. For example, this approach can be used for multicomponent determination of several dozen endogenous steroids [95]. Hill and co-workers (2019) developed and validated the GC–MS/MS method for the quantification of 58 unconjugated steroids and 42 polar steroid conjugates, including neuroactive or immunomodulatory steroids. Such steroid profiling in male and female blood samples, including the blood of pregnant women and the umbilical cord, can be useful in quickly diagnosing various pathologies, identifying their causes, or seeking new therapy options.

Other studies also used the GC–MS/MS for analysis of circulating steroids in humans or other vertebrates [91,92]. Hansen and co-workers (2011) described an optimized and validated GC–MS/MS method for determining pregnenolone, progesterone, DHEA, androstenedione, testosterone, 5α-dihydrotestosterone, estrone, 17α-estradiol, and 17β-estradiol in blood plasma and serum of certain vertebrates [91]. Moreover, the triple quadrupole with EI operating in selected reaction monitoring (SRM) mode allowed extremely low background noise when analyzing biological samples. Applying different MS analyzers, such as ion traps, the use of a scan mode enabling MS/MS monitoring is also possible [96,97].

Finally, Kanceva and co-workers (2015) used a GC–MS system to study the relationship between levels of certain steroids and multiple sclerosis, one of the most common neurological diseases [88]. For this purpose, they analyzed steroids and polar conjugates of steroids (51 in total) in 12 patients with multiple sclerosis who were untreated with steroids, and 6 women as a control group. In patients with multiple sclerosis, they observed a significant increase in circulating levels of C21 steroids (e.g., pregnenolone), their polar conjugates (e.g., pregnenolone sulfate), and some bioactive C19 steroids (e.g., androstenedione). This work shows the importance of simultaneous targeted profiling of a broad spectrum of steroids using GC–MS methods. An accurate determination of circulating hormone levels can help us understand their effect on nervous system functions, for example, on their disruption of the balance between neuroprotection and excitotoxicity.

### 4.3. Liquid Chromatography–Mass Spectrometry

#### 4.3.1. Introduction and History of LC–MS

The combination of LC or supercritical fluid chromatography (SFC) with MS took place a few decades later (the 1980s) than GC–MS due to demanding technical requirements [68]. In the field of steroid analysis, the LC–MS technique has significantly expanded in recent years, mainly because it allows high sample throughput to be achieved [18]. Although this technique is suitable for a quick targeted analysis of conjugated and unconjugated steroids, its use in untargeted approaches is less appropriate. Due to existing factors that can significantly impair its specificity, the LC–MS tool in still used less frequently in untargeted analysis (e.g., lower chromatographic resolution compared to GC, higher susceptibility of soft ionization to matrix effects compared to EI).

The abovementioned SFC is not a new separation technique because it has existed since the 1960s [98,99]. Technological progress and the introduction of commercial ultra-high-performance supercritical fluid chromatography (UHPSFC) tools at the beginning of the millennium contributed to its more comprehensive application [68,98,99]. Many benefits are associated with SFC, such as a reduction in the consumption of harmful organic solvents (so-called green analytical chemistry), the robustness of advanced techniques, reproducibility, selectivity, sensitivity, and its usefulness in the separation of thermally unstable, nonvolatile, and chiral compounds [99]. Compared to other separation techniques, better separation of enantiomers and isomers can be achieved with SFC [100]. Due to the possibilities offered by commercial UHPSFC systems, both in the choice of different organic modifiers and types of stationary phases, this approach is highly versatile and selective [98]. These properties make UHPSFC useful for the separation of steroids, in addition to other compounds sharing similar structures and mass spectra.

#### 4.3.2. Liquid Chromatography

**Figure 3:**
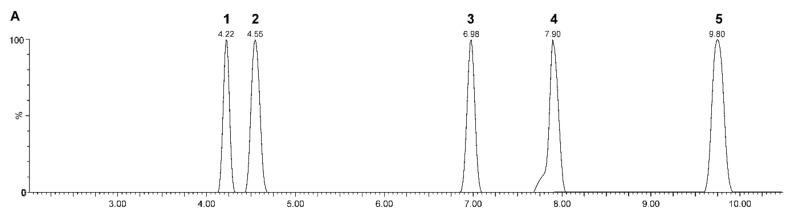
Reversed-phase chromatographic separation (Kinetex Biphenyl column, 2.1 × 100 mm; 1.7 μm) of selected neuroactive steroids by ultra-high-performance liquid chromatography–tandem mass spectrometry (UHPLC–MS/MS). Figure 3 shows the merged chromatograms for the steroid standards mixture: 1—dehydroepiandrosterone, 2—testosterone, 3—pregnenolone, 4—allopregnanolone, and 5—progesterone. Retention times (min) are indicated above the peak.

Reverse phase (RP) chromatographic separation is widely used in steroid analysis [79] (Figure 3 shows an example). Compared to the normal phase, RP chromatographic separation is more effective due to the hydrophobicity of unconjugated steroids [65]. The best tool for separating a number of steroids is elution in gradient mode [79]. Compared to GC–MS, LC–MS achieves lower chromatographic resolution [33,81]. Better chromatographic efficiency and sensitivity can be achieved using columns with particles smaller than 2 μm [101]. The use of such small particles is associated with high back pressures and has become possible by the introduction of ultra-high-performance liquid chromatography (UHPLC) technology that is able to withstand them [102]. Another option offering similar chromatographic separation, but at lower pressures and without the need for UHPLC instrumentation, is the particle-packed columns based on so-called fused core particle technology. This alternative can have almost the same efficiency as the abovementioned particles, but they are larger compared to them and, therefore, no high back pressures are generated [101]. This makes this technology compatible with conventional high-performance liquid chromatography (HPLC) systems.

#### 4.3.3. Ionization Techniques Used in LC–MS

The first important step in connecting LC and MS was the introduction of atmospheric pressure ionization techniques (APIs) [103]. Because LC–MS methods are API-compatible, they can be used for analysis of intact conjugates [48]. Commonly used ionization techniques for LC–MS detection of steroid substances are electrospray ionization (ESI), atmospheric-pressure chemical ionization (APCI), and atmospheric-pressure photoionization (APPI) [97]. ESI is also suitable for the analysis of polar molecules and steroid conjugates. When analyzing less polar substances, chemical modification may be required to increase ESI sensitivity to the level of APPI and APCI ionization techniques. APPI and APCI ionization techniques are more suitable for unconjugated nonderivatized steroids. Moreover, APCI provides more selective ionization and lower matrix effects for some substances [102].

#### 4.3.4. Matrix Effects

In connection with LC–MS analysis, so-called matrix effects are often mentioned [104]. Although the LC–MS/MS system is highly selective and sensitive, matrix effects are its biggest problem [101]. A common cause of matrix effects is compounds being present in the matrix that co-elute with the analytes and disrupt their ionization [105]. Changing ionization efficiency can lead to ion suppression or ion enhancement. The ionization process may be disrupted by both organic and inorganic substances of endogenous origin which come from the sample and are, therefore, present in the final extract (e.g., salts, urea, lipids, and peptides), but also substances of exogenous origin, i.e., those that enter the sample during the preparation process [106]. The biggest problem in this respect is the analysis of extracts of complex matrices using the ESI ionization technique [105]. APPI and APCI techniques are less susceptible to matrix effects. As a result, co-elution of the analyte with interfering components can lead to a negative impact on the accuracy, precision, and sensitivity of the LC–MS method [101]. To eliminate, reduce, or at least compensate for matrix effects, it may be beneficial to pay attention to the adjustment of the sample’s quantity, the preparation of the sample, the modification of chromatography conditions (e.g., optimization of the mobile phase, change of column parameters), the optimization of MS (choice of ionization technique, the polarity of ionization), and the selection of possible calibration techniques (e.g., external matrix-matched calibrators, internal standards, and standard addition).

#### 4.3.5. Sample Preparation for LC–MS

In methods based on LC–MS, preparation of the sample before analysis is essential [102]. The most common techniques used for sample preparation include protein precipitation, solid-phase extraction (SPE), and liquid–liquid extraction (LLE). Extraction of steroids from biological matrices, such as blood plasma, serum, urine, or saliva, is performed in order to remove interfering substances from the samples, increase the sensitivity of the methods, and protect the instrumentation, and, in particular, to extend the life of the chromatographic columns [107]. Due to the precipitation of proteins with methanol or acetonitrile, the steroids are released from binding to their carrier proteins.

The offline SPE allows for the removal of interfering substances of the matrix and concentrates the analytes [29]. Naldi and co-workers (2016) designed and validated a fully automated method combining an online SPE with LC–MS/MS for simultaneous analysis of the free and conjugated forms of selected steroids in urine and various types of water (sewage water, river water, etc.). A similar arrangement may be seen in works concerned with the analysis of other matrices; for example, human saliva or plasma [53,108]. Online SPE has several advantages compared to the offline approach [29,53]. These include working with smaller sample volumes, reduced risk of procedural errors, better repeatability and reproducibility, reduced sample preparation time, and increased sample throughput. The development of online SPE, however, is not always easy and may be associated with a number of problems, such as the incompatibility of SPE sorbents and analytical column and broadening of peaks. Li and co-workers (2018) developed a new method for quick, highly sensitive, specific, and simultaneous determination of estrone, estradiol, and estriol in human saliva [21]. This method combines LC–MS/MS with a miniaturized and high-throughput SPE on a hydrophilic-lipophilic-balanced microplate with 96 wells. Miniaturized SPE allows the purification of samples and analyte enrichment without the need for derivatization, evaporation, or LLE. Advantages of this approach are also reduction in solution consumption and manual handling time. The sensitivity of this method is 1 pg.mL^−1^ and it allows the quantification of trace estrogen levels.

#### 4.3.6. LC–MS Applications in Steroid Analysis

In recent years, LC–MS/MS has become the main technique for the analysis of steroid hormones [33]. The progressive improvement of LC systems and the introduction of MS/MS led to gradual replacement of the GC–MS techniques [51]. The clear advantages of LC–MS/MS include the speed of analysis, specificity, possibility of automation, and easy and time-saving sample preparation [18,33,102]. For high sample throughput and fast processing time, the LC–MS/MS methods are particularly attractive for clinical laboratories [51]. However, the expansion of LC–MS/MS systems and their routine use are complicated mainly by high acquisition costs and higher technical complexity [102]. No hydrolysis of conjugates or chemical derivatization are required [33]. Compared to IA, the determination of steroid hormones using LC–MS/MS requires smaller sample volumes [102]. Furthermore, chemical derivatization can improve ionization, increase sensitivity, and, consequently, reduce the limits of quantifications (LOQs) [19]. Including the derivatization step may also help in determining the location of functional groups on the steroid molecule because derivatization usually supports unique MS fragmentation [33].

MS/MS-based analysis achieves higher selectivity and sensitivity than using only a single mass analyzer [104,107]. One of the most common tandem mass analyzers is the triple quadrupole. This instrument is the most widely used for steroid hormone quantification due to its versatility and sensitivity. The triple quadrupole allows different working modes, such as full scan and product ion mode. [104]. The scanning mode is referred to as an SRM or MRM transition, in which only pre-selected ions are detected. The MRM mode allows the simultaneous quantification of several analytes in a single experiment. Other compounds (unselected analytes) presenting a chemical background of the sample are not detected, which is the biggest disadvantage of this approach [68]. The MRM mode provides the highest sensitivity and selectivity with regard to these favorable qualities [104]. This mode is one of the most reliable tools for confirming the presence of specific compounds in samples. The SIM mode can be also used; however, it is characterized by lower sensitivity and selectivity. A combination of LC methods and triple quadrupole MS/MS can provide qualitative and quantitative information for many analytes in the same sample (multiplexing) and in one analytical run [107].

Keevil and co-workers (2017) used an LC–MS/MS instrument operating in positive ionization mode to analyze testosterone in saliva [24]. Sample preparation included the addition of an internal standard and LLE using methyl-tert-butyl ether. This analytical tool is more specific and sensitive compared to IA. The UHPLC–MS/MS system was also used to determine neurosteroids and steroids with immunomodulatory effects in human CSF and blood plasma [32]. MS detection was performed using triple quadrupole-MS with positive ESI working in MRM mode. In this study, free DHEA, its selected metabolites, namely 7α-hydroxy- and 7β-hydroxy-DHEA, 7-oxo-DHEA, and 16α-hydroxy-DHEA, in addition to cortisol and cortisone, were quantified to better understand degenerative diseases and, in particular, to monitor their development and progression. Furthermore, Caruso and co-workers (2014) analyzed NASs in blood plasma and CSF by UHPLC–MS/MS with APCI ionization working in the positive mode [12]. The study showed differences in levels of steroid hormones between 26 men diagnosed with multiple sclerosis (avg. age of 34 years), specifically in the relapsing-remitting form, and in 12 samples representing the control group (avg. age of 29 years). For example, pregnenolone, progesterone, and 5α-dihydrotestosterone levels were increased, and in 5α-dihydroprogesterone and allopregnanolone, levels were decreased in patients with multiple sclerosis compared to the control group. In another study, chromatographic separation of the samples performed on a PR-C18 column was combined with an MS/MS analyzer using the positive mode of APCI and SRM transitions [109]. A simple and specific method has been developed for simultaneous determination of selected NASs, namely cortisone, cortisol, DHEA, estradiol, progesterone, pregnenolone, and testosterone. Sample preparation prior to analysis employed LLE using an ethyl acetate extraction procedure for the seven NASs from blood plasma and brain tissue of laboratory rats. The results suggest that the observed differences in the levels of some endogenous NASs may have potential as biomarkers usable for the diagnosis or treatment of depression.

The analysis of steroid substances is not limited only to blood serum and plasma, and other matrices are also used. Nguyen and co-workers (2011) developed and validated a method for the simultaneous quantification of several estrogen hormones, namely estriol, estrone, 17α-estradiol, and 17β-estradiol in human CSF, based on the heart-cutting two-dimensional LC–MS/MS system [36]. The sample preparation included LLE with determined extraction recoveries between 91% and 104%, and subsequent derivatization with dansyl chloride. By including the derivatization step, an increase in sensitivity limits was achieved. The accuracy and precision of this method was more than 86% for 17β-estradiol and 17α-estradiol and 79% for estriol and estrone. However, to improve detection limits, the ion trap instrument used should be replaced with a more sensitive means of detection, such as a triple quadrupole. Finally, the developed method was applied to analyze estrogen in patients suffering from ischemic trauma.

Gao and co-workers (2015) developed a highly sensitive, selective, and fast online SPE LC–MS/MS method to determine estradiol and some other steroid hormones (cortisol, cortisone, testosterone, progesterone, corticosterone, DHEA) [53]. All analyzed hormones, except for DHEA, had a LOQ lower or equal to 5 pg.mL^−1^. The LOQ for DHEA was 10 pg.mL^−1^. The authors finally used this method to determine selected steroid hormones in saliva samples, in which no estradiol levels were detected when using a routine IA.

#### 4.3.7. SFC–MS Applications in Steroid Analysis

One of the first supercritical fluid chromatography–mass spectrometry (SFC–MS) applications in steroid analysis is associated with the end of the last century [110]. Tuomola and co-workers (1998) applied a packed column SFC combined with APCI–MS to determine androstenone in porcine fat. Fat sample processing was simple and included only dichloromethane extraction. Xu and co-workers (2006) described a method for the separation and quantification of 15 structure-related estrogen metabolites (e.g., estrone, estradiol, estriol, 4-methoxyestron, 2-hydroxyestradiol) by a packed column using SFC–MS/MS in less than 10 min [111]. Chromatographic separation was performed on a cyanopropyl silica column connected in series with a diol column. The mobile phase was carbon dioxide with a linear gradient of methanol. This analytical approach is several times faster compared to RP–HPLC–MS/MS analysis of the same group of analytes. The SFC–MS is therefore more suitable for the analysis of larger sets of samples. Furthermore, Doué and co-workers (2015) developed and validated UHPSFC–MS/MS for analysis of several conjugated urinary steroids, specifically glucuronide and sulfate steroids in bovine urine samples [112]. Their analysis is a suitable tool for the monitoring of anabolic steroid misuse (e.g., food industry, doping). The optimization of several SFC conditions (e.g., stationary phase, addition of modifiers, back pressure, column temperature) resulted in 2 different approaches enabling the analysis of 8 glucuronide and 10 sulfate steroids. Furthermore, UHPSFC–MS/MS provided better sensitivity and repeatability in less run time.

Other studies also used SFC–MS for analysis of steroid substances [100,113,114]. Kock and co-workers (2018) described a novel UHPSFC–MS/MS method with positive ESI for simultaneous determination of 19 steroids (from androgen, estrogen, progestogen, and glucocorticoid classes) in human plasma within 5 min [100]. Other biological materials, such as CSF or urine, are applicable for the profiling of endogenous steroids or the screening of doping agents, respectively [113,114].

#### 4.3.8. Ion Mobility

Among steroids, a large number of isomers and isobaric compounds with different biological effects can be found [115]. A common problem with LC methods is their limited resolution of structurally similar substances [116]. To achieve their separation, it is necessary to extend the run time of chromatographic separation. Some isomers, especially stereoisomers, may be subject to similar fragmentations, complicating the rapid identification and quantification of steroids by MS/MS. An additional dimension of separation can be provided by complementing the MS system with ion mobility spectrometry (IMS), which allows further differentiation of isomers or isobaric compounds [68]. IMS can be easily combined with existing GC–MS or LC–MS methods [116]. In IMS–MS techniques, time separators such as drift tube ion mobility spectrometry (DTIMS), traveling wave ion mobility spectrometry (TWIMS), and spatial separators, such as differential ion mobility spectrometry (DMS), also referred to as high-field asymmetric ion mobility spectrometry (FAIMS), are most commonly used for steroid analysis [117].

According to the study of Chouinard and co-workers (2017), DTIMS holds considerable potential for improving the analysis of isomer forms of steroids [116]. Rister and Dodds (2020a) used the LC–MS system containing TWIMS for the analysis of steroid hormone isomers [115]. The results showed that a combination of LC and IMS–MS systems can lead to an increase in the resolution of steroid isomers compared to using only LC or IMS. Applying LC–IMS–MS can also achieve faster analysis of steroid isomers compared to simple LC–MS. Moreover, IMS also provides a CCS (ion-neutral collision cross-sections) parameter that can be used together with retention time and *m/z* as an adjunct in identifying analytes.

Finally, the use of analytical techniques combining the LC–MS/MS system with the DMS also enhance the performance of the determination of endogenous steroids in human blood serum and plasma [118]. The inclusion of DMS has led to an increase in the specificity of the analysis, which makes it possible to simplify sample preparation, reduce chromatographic separation time, and increase analysis speed. Ray and co-workers (2015) developed and validated a highly sensitive and specific method for determining corticosterone, 11-deoxycortisole, 11-deoxycorticosterone, 17-hydroxyprogesterone, and progesterone. Because the pairs corticosterone and 11-deoxycortisole, and 11-deoxycorticosterone and 17-hydroxyprogesterone, are isomer pairs, their distinction by LC–MS/MS is complicated due to similar fragmentation and chromatographic retentions. Combining chromatographic separation and DMS increased isomer resolution and reduced background noise.

Recently, the use of IMS as a single separation technique for the analysis of steroids without the inclusion of chromatographic separation has been the subject of interest [117]. Analysis of steroids using IMS–MS without chromatography would significantly reduce the time of acquisition and sample preparation.

### 4.4. Metabolomics, Targeted and Untargeted Mass Spectrometry Analysis

In recent years, the field of metabolomics has been of great interest, and has helped us in further understanding metabolic mechanisms under physiological and pathological conditions [119]. This field focuses on comprehensive analysis of intracellular and extracellular metabolites in biological fluids, cells, tissues, and organisms [72,120]. Studying only a few steroids, or a comprehensive monitoring of steroid metabolome (so-called steroidome), can lead to the discovery of new steroids, steroid pathways or biological markers that may be useful for diagnosis, monitoring, prevention, or prediction of disease risk, as well as drug development [119,121]. Steroid metabolome studies may result in the development of more sophisticated approaches to screening or diagnosing a number of endocrine diseases [122,123]. Monitoring differences in steroidome in healthy subjects and patients may contribute to the discovery of candidate steroid biomarkers for schizophrenia, but also for other psychiatric disorders (e.g., mood, anxiety disorders) [124,125,126,127]. These findings can, of course, improve the quality of life of patients, because changes in the level of metabolites are often associated with a number of diseases and often occur before the clinical manifestation of a disease [72].

However, the analysis of steroid profiles with chromatography techniques, coupled with MS, is an analytical challenge due their large dynamic range, their extraction from complex biological matrices, or the selectivity of the analytical techniques [121]. Due to the large variability of metabolites in terms of their chemical diversity, polarity, molecular weight, and concentration range, a single analytical tool and sample preparation protocol cannot be used within the framework of an untargeted approach, because no sampling strategy or analytical technique can cover all the metabolites present [81]. By contrast, targeted analysis, in which specific groups of metabolites are analyzed, is often sufficient with a single strategy. Targeted analysis is carried out based on a certain hypothesis and focuses on predefined analytes; in contrast, untargeted analysis is global and does not focus on specific analytes or hypotheses [80]. Both approaches can be combined.

In their paper, Palermo and co-workers (2017) presented an untargeted metabolomics approach based on UHPLC–MS/MS for the study of the urinary steroidal profile [128]. This proposed workflow is able to detect up to 3000 metabolites of steroid origin using high-resolution mass spectrometry. The study of urinary steroids is an approach that can be used to monitor various pathological conditions and to detect the illicit use of anabolic steroids. Targeted and untargeted approaches can be combined, and they can provide a more comprehensive view of the issue [129]. An example is the isotope dilution-based targeted and untargeted profiling of carbonyl neurosteroids and steroids. This hybrid method allows absolute quantification of pregnenolone, progesterone, 5α-dihydroprogesterone, 3α,5α-tetrahydroprogesterone, and 3β,5α-tetrahydroprogesterone, and relative quantification of other carbonyl-containing steroids in animal models.

In-depth views of the different aspects of steroidomics can be found in a number of existing publications [121,130,131].

### 4.5. Validation of Bioanalytical Method

Validation of the method should demonstrate that the method is sufficiently reliable for determining the selected analyte in a particular biological matrix [132]. According to the European Medicines Agency (EMA) guideline, the validation of a method should include, for example, the determination of calibration range, accuracy, precision, and matrix effect. Method validation can also be carried out based on the Food and Drug Administration (FDA) guidelines [133].

## 5. Conclusions

Analytic methods summarized in Table 1 allow the monitoring of the differences between the levels of neuroactive steroids in different physiological conditions and pathological conditions. They may represent a useful instrument in deepening knowledge of physiological mechanisms and pathophysiology of some diseases. Monitoring changes in steroid hormone levels can be an effective tool in the search for new biological markers useful in monitoring, preventing, or predicting disease risks. The knowledge gained from metabolite studies can also increase our understanding of the pathophysiology of certain diseases and provide new insights into the possibilities of their diagnosis, or even treatment, which will contribute, for example, to the development of new drugs and procedures. All of the steroid analysis presented herein, whether methods based on immunoassay or mass spectrometry, have their advantages and disadvantages. It is especially important to know the virtues but also the limits of such analytical methods, and to consider their use for the intended purposes, accordingly, to obtain reliable results. Individual approaches to the analysis of steroids may complement each other, thus providing specific pieces of information and allowing us to compile a comprehensive picture of the issue.

## Figures and Tables

**Table 1:** Summary of selected analytical approaches used for neuroactive steroids analyses. IA: immunoassay; LC–MS(/MS): liquid chromatography–(tandem) mass spectrometry; SFC–MS(/MS): supecritical fluid chromatography–(tandem) mass spectrometry; gas chromatography–(tandem) mass spectrometry; IMS: ion mobility spectrometry; CSF: cerebrospinal fluid.

Analytical Method	Sample Type	Class of Analytes	Reference
IA	saliva, serum	glucocorticoids, mineralocorticoids	[22]
	serum, CSF	androgens, progestins, estrogens	[35]
	serum	androgens	[39]
	plasma	estrogens	[44]
	plasma, serum	androgens, progestins, estrogens, glucocorticoids	[54]
	serum	androgens	[55]
	serum	androgens	[56]
	plasma	androgens, estrogens	[57]
	serum	androgens, estrogens	[62]
	urine	estrogens	[63]
	serum	androgens, estrogens	[64]
	serum	androgens	[67]
	serum, CSF	progestins, glucocorticoids	[89]
	plasma	progestins, estrogens, glucocorticoids	[126]
GC–MS(/MS)	serum	estrogens	[25]
	urine	androgens, progestins, glucocorticoids	[27]
	serum	androgens, estrogens	[62]
	serum	androgens, progestins, estrogens	[66]
	serum	androgens	[67]
	feces	neutral fecal steroids	[76]
	serum	androgens, progestins, estrogens	[88]
	serum, CSF	androgens, progestins	[89]
	plasma, serum	androgens, progestins, estrogens	[91]
	plasma	androgens, progestins	[92]
	plasma	androgens, progestins, estrogens	[94]
	serum	androgens, progestins, estrogens, glucocorticoids	[95]
	serum	androgens, progestins, glucocorticoids	[125]
	plasma	androgens, progestins, estrogens	[126]
LC–MS(/MS)	serum	androgens, progestins, glucocorticoids	[4]
	plasma, CSF	androgens, progestins, estrogens	[12]
	saliva	estrogens	[21]
	saliva	androgens	[24]
	scalp hair	androgens, progestins, glucocorticoids	[26]
	finger nails	androgens, progestins, glucocorticoids, mineralocorticoids	[28]
	water matrices, urine	estrogens	[29]
	urine	androgens	[30]
	urine	androgens	[31]
	plasma, CSF	androgens, glucocorticoids	[32]
	CSF	estrogens	[36]
	serum	androgens, progestins, glucocorticoids	[37]
	plasma	androgens, progestins, glucocorticoids	[38]
	saliva	androgens, progestins, estrogens, glucocorticoids	[53]
	urine	estrogens	[63]
	serum	androgens, progestins, estrogens	[84]
	serum	progestins, androgens	[85]
	serum	androgens, progestins, glucocorticoids, mineralocorticoids	[108]
	plasma, brain tissue	androgens, progestins, estrogens, glucocorticoids	[109]
	plasma	androgens, progestins, estrogens, glucocorticoids, mineralocorticoids	[123]
	urine	>3000 individual metabolic features	[128]
	brain tissue	carbonyl steroids	[129]
SFC–MS(/MS)	plasma	androgens, progestins, estrogens, glucocorticoids, mineralocorticoids	[100]
	fat	androgens	[110]
	urine, serum	estrogens	[111]
	urine	androgens, estrogens	[112]
	CSF	androgens, progestins, estrogens, glucocorticoids	[113]
	urine	androgens	[114]
(LC–)IMS–MS(/MS)	standard solutions	androgens, glucocorticoids, mineralocorticoids	[115]
	standard solutions	androgens, progestins, estrogens, glucocorticoids, mineralocorticoids	[116]
	serum, plasma	progestins, glucocorticoids	[118]
